# Analysis of the heavy metal contents’ effect on steroidal saponins and the anti-breast cancer activity of *Paris polyphylla* var. *yunnanensis*

**DOI:** 10.3389/fphar.2023.1277395

**Published:** 2023-10-26

**Authors:** Hai-Ling Li, Cui-Ping Yan, Jun-Sheng Qi, Shuo Zhang, Dong-Qin Guo, Wen-Chao Gu, Ying-Mei Wu, Yu Wu, Nong Zhou

**Affiliations:** ^1^ Chongqing Engineering Laboratory of Green Planting and Deep Processing of Famous-Region Drug in the Three Gorges Reservoir Region, College of Biology and Food Engineering, Chongqing Three Gorges University, Chongqing, China; ^2^ College of Pharmacy, Nanjing University of Chinese Medicine, Nanjing, China; ^3^ Taizhou Institute for Drug Control, Taizhou, China; ^4^ Nantong Hospital Affiliated to Nanjing University of Chinese Medicine, Nantong, China; ^5^ College of Pharmacy, Dali University, Dali, China

**Keywords:** heavy metal, steroidal saponins, breast cancer, *Paris polyphylla* var. *yunnanensis*, interrelationships

## Abstract

**Background:** *P. polyphylla* var. *yunnanensis*, as a near-threatened and ethnic medicine in China, used to be a key ingredient in traditional Chinese medicine in treatment of traumatic injuries, sore throat, snakebites, and convulsions for thousands of years. However, there were no reports on the inverse relationship between the contents of heavy metals and saponins and its anti-breast cancer pharmacological activity in *P. polyphylla* var. *yunnanensis*.

**Methods:** The present study aimed to reveal the characteristics of heavy metal contents and saponins and its anti-breast cancer pharmacological activity and their interrelationships in *P. polyphylla* var. *yunnanensis* from different production areas. The contents of heavy metal and steroidal saponins in *P. polyphylla* var. *yunnanensis* were analyzed by inductively coupled plasma mass spectrometry (ICP-MS) and the high-performance liquid chromatography technique, respectively. The Pearson correlation was used to study the correlation between saponins and heavy metals. 4T1 mouse mammary tumor cells were selected and cultivated for antitumor studies *in vitro*. Cell Counting Kit-8 (CCK-8) assay, Hoechst staining, and flow cytometry analysis were used for the examination of the proliferation and apoptosis of 4T1 tumor cells. Mouse breast cancer 4T1 cells were subcutaneously injected into BALB/c mice to construct a tumor model to explore the *in vivo* inhibitory effect on breast cancer. TUNEL assay and immunohistochemistry were used for the examination of the effect of *P. polyphylla* var. *yunnanensis* from different origins on cancer cell proliferation and apoptosis induction in 4T1 tumor mice.

**Results:** Heavy metal contents were highly correlated with the content of steroidal saponins. The overall content of 10 metals in the three producing origins was of the order C3 >C2 >C1. The total content of eight steroidal saponins in the extracts of *P. polyphylla* var. *yunnanensis* from three different origins was C1 >C2 >C3. The Pearson correlation study showed that in all of the heavy metals, the contents of Cd and Ba were positively correlated with the main steroidal saponins in *P. polyphylla* var. *yunnanensis*, while Al, Cr, Cu, Fe, Zn, As, Hg, and Pb showed a negative correlation. *In vitro* experiments showed that the extracts of *P. polyphylla* var. *yunnanensis* from three origins could inhibit the proliferation and induce cell apoptosis of 4T1 cells in a concentration- and time-dependent manner, especially in the C1 origin. *In vivo* experiments showed that the extract of *P. polyphylla* var. *yunnanensis* from the three origins could inhibit the growth of tumors and induce the apoptosis of tumor cells. In the three origins, C1 origin had the lowest total heavy metal level but the highest total steroidal saponin level. Therefore, it showed a better effect in reducing the expression of the human epidermal growth factor receptor 2 (HER2) and Kiel 67 (Ki67) and increasing the expression of p53 in tumor tissues compared to the other origins. In conclusion, in the three origins, C1 origin exhibits antitumor pharmacological effects *in vivo* and *in vitro* which are better than those in the other origins.

**Conclusion:** In this study, we found that with the increase of the heavy metal content, the content of steroid saponins and anti-breast cancer activity decreased. The results showed that the high content of the total heavy metals may not be conducive to the accumulation of steroidal saponins in *P. polyphylla* var. *yunnanensis* and lead to the low anti-breast cancer activity. The results of this study suggest that the content of heavy metals should be controlled in the artificial cultivation process of *P. polyphylla* var. *yunnanensis*.

## 1 Introduction

As a plant belonging to the Liliaceae family, *Paris polyphylla* var. *yunnanensis* (Franch.) Hand.-Mazz. (*P. polyphylla* var. *yunnanensis*) has long been used as a key ingredient in traditional Chinese medicine to treat traumatic injuries, sore throat, snakebites, and convulsions (Chinese Pharmacopoeia Commission, 2020). Its medicinal use was first referred to in《Shen Nong Ben Cao Jing》. In recent years, *P. polyphylla* var. *yunnanensis* has been applied as the main raw material to produce these famous Chinese patent medicines, such as Gongxuening capsules, Jidesheng Sheyao tablets, and antiviral granules (Wu, et al., 2012; Qin et al., 2013; Wen et al., 2015). The main active components of *P. polyphylla* var. *yunnanensis* include steroid saponins, cholestanols, flavonoids, and triterpenes, among which steroidal saponins account for approximately 80% of the total content (Wang et al., 2010; Kang et al., 2012a; Kang et al., 2012b; Shah et al., 2012; Negi et al., 2014). It has now been demonstrated that steroidal saponins possess various pharmacological properties, including antitumor effects (Sun et al., 2014; Lai et al., 2021; Li et al., 2021; Liu et al., 2022) against breast cancer, liver cancer, esophageal cancer, ovarian cancer, leukemia, colorectal cancer, and hepatocellular carcinoma (Sun et al., 2011; Wang et al., 2016; Chen et al., 2019).

Contamination of heavy metals in the environment due to diverse human activities is a serious environmental issue that cannot be ignored (Hussain et al., 2019; Farooq et al., 2020). In fact, heavy metal is one of the most significant influencing factors for the accumulation of active components during plant growth because it could be absorbed and enriched by the plant to participate in the synthesis of active components as reaction catalysts or affect the expression of active component synthetase genes (Yang et al., 2014; Ullah and Alqahtani, 2022). The biosynthesis of secondary metabolites may eventually be impacted by the cultivation of medicinal plants in heavy metal-contaminated environments, leading to dramatic alterations in the quantity and quality of these substances. In recent years, extensive research has been conducted to reveal that fungi play an important role in the contents and properties of *P. polyphylla* var. *yunnanensis* (Huang et al., 2009; Liu et al., 2017; Li et al., 2018). However, there are still few studies focusing on the heavy metals present in *P. polyphylla* var. *yunnanensis*, which greatly affects the safety and effectiveness. It is hypothesized that heavy metal pollution may affect the quality of the medicinal materials, mainly by affecting the accumulation of its active ingredients and pharmacological activity. Therefore, the correlation between steroid saponin contents, pharmacological activity, and heavy metal content of *P. polyphylla* var. *yunnanensis* was explored in this study.

In this study, the presence of heavy metals is detected in three areas of *P. polyphylla* var. *yunnanensis*, with the same duration of growth, and that of steroidal saponins was found in plants. Furthermore, the correlation between the heavy metal and steroidal saponin contents was analyzed. The experimental research on anti-breast cancer activity was carried out to reveal heavy metal contents, steroidal saponins, and its antitumor pharmacological activity and their interrelationships in *P. polyphylla* var. *yunnanensis* from different production areas.

## 2 Materials and methods

### 2.1 Reagents and extraction

The raw *P. polyphylla* var. *yunnanensis* rhizome was collected in three different areas across Yunnan province, China, of which C1 was obtained from Laoyutun Village, Yanglin Town, Songming County, Kunming City; C2 was obtained from Ahaizhai Village, Shanyang Town, Yongping County; C3 obtained from Yuhu Village, Baisha Town, Yulong County, Lijiang City. First, 40 g of the *P. polyphylla* var. *yunnanensis* rhizome was weighed and passed through sieve no. 6. Approximately 400 mL of 80% ethanol was added, and reflux extraction was performed three times. All of the extraction solution was filtered to a concentrate. Subsequently, the extract was freeze-dried and refrigerated at −20°C for later use.

### 2.2 Determination of heavy metal content by ICP-MS

Digestion of the medicinal material: 0.20 g of the *P. polyphylla* var. *yunnanensis* dry plant sample powder was weighed and added into a polytetrafluoroethylene digestion tank. Then, add 8 mL of concentrated nitric acid; seal; cover it up with the digestion lid; leave it overnight; and place it in the microwave digestion apparatus. The digestion process is as follows: first, it was heated up from room temperature to 140°C for 6 min and maintained for 2 min. Second, it was heated up to 160°C at the rate of 10°C·min^−1^ for 5 min. Third, it was heated up to 180°C at the rate of 15°C·min^-1^ for 15 min and finally held at 50°C for 10 min. After the digestion was complete, the digestion tank was taken out, and the acid was dried at 120°C in a ventilator, transferred to a 50-mL volumetric bottle with deionized ultra-pure water, and then shaken for measurement. Approximately 8 mL of concentrated nitric acid and 1 mL of hydrofluoric acid were added into the polytetrafluoroethylene digestion tank for preparation of a blank solution through the same method.

ICP-MS parameters are as follows: a power of 1.4 kW, an atomizing gas flow of 1.224 L min^-1^, an auxiliary gas flow of 1.0 L min^-1^, a cooling gas flow of 14.0 L min^-1^, an atomizing chamber temperature of 2.0°C, a peristaltic pump speed of 30r·min^-1^, a sampling depth of 3.91 mm, 10 times of scanning, and a 26-s length of analysis. The AFS parameters are as follows: a mercury lamp, a negative pressure of 290 V, a carrier gas flow of 400 mL min^-1^, a shielding flow of 1,000 L·min^−1^, a lamp current of 10 mA repeated three times, and a 10-s reading time. With Ge, Rh, and Bi as internal standard elements, the contents of Al, Cr, Fe, Cu, Zn, Ba, Pb, As, Cd and Hg were determined by ICP-MS.

### 2.3 Determination of steroidal saponin content by HPLC

Approximately 30 mg of the rhizome extract from *P. polyphylla* var. *yunnanensis* was weighed in a 10-mL volumetric bottle, subjected to ultrasonic treatment for 30 min, and filtered with a 0.22-um microporous membrane. The steroidal saponin content was determined with the following chromatographic conditions: The chromatographic column used was Accucore PFP C_18_ (2.1 mm × 100 mm; 2.6 μm). The mobile phase was acetonitrile (A)–water (B), gradient elution: 0–5 min and 20%–36% A; 5–17 min and 36% A; 7–12 min and 36%–37% A; 12–15 min and 37%–39% A; and 15–17 min and 39%–45% A. The detection wavelength was 203 nm, the flow rate was 0.2 mL·min^−1^, and the injection volume was 5 μL. The column temperature was 30°C, and the sample room temperature was 10°C.

### 2.4 Cell lines and cell culture

Purchased from Shanghai Zhong Qiao Xin Zhou Biotechnology Co., Ltd., 4T1 breast cancer cells were cultured in the RPMI-1640 medium covered with the bottom of T-25 culture flasks. The flasks were placed in an incubator with 5% CO_2_ at 37°C for axenic cultivation. During incubation, the cell morphology was photographed using an inverted microscope every 8 h for the observation of cell growth. After digestion, centrifugation, discarding of the supernatant, and the resuspension of cells with the appropriate culture medium, stained with 4% Trypan Blue solution (mixed at 9:1 ratio) for 3 min, the cell counts were performed.

### 2.5 CCK-8 assay

CCK-8 was used to evaluate the level of viability. The cells at a density of 4 × 10^4^ cells/mL (100/µL) were seeded in multiple 96-well plates and incubated for 24 h under the standard atmosphere with 5% CO_2_ at 37°C. *P. polyphylla* var. *yunnanensis* extracted from the three origins were set to five dosage concentrations (11.25, 22.5, 45, 90, and 180 µM) using 10% bovine serum medium and the multiple dilution method. After the cells adhered to the wall, the cell culture medium was replaced with the extract solution. Meanwhile, the groups containing only the culture medium was set as the blank control. There were three parallel groups set for each concentration. With each group exposed to the reagent for 24 or 48 h, the optical density (OD) was measured at 450 nm by a microplate reader. The cell viability ratio was calculated using the following equation:

$$\text{Proliferation rate}\left(\%\right)=\left({\text{OD}}_{\text{test}}-{\text{OD}}_{\text{control}}\right)/\left({\text{OD}}_{\text{control}}-{\text{OD}}_{\text{conrtol}}\right)\times 100\%.$$

### 2.6 Hoechst staining

Hoechst staining refers to a DNA-specific dyeing method used to assess cell apoptosis. After being collected at a suspension density of 2×10^5^ cells/mL, the 4T1 cells at the mid-log phase with a good status of growth were seeded in 12-well plates. After 24 h of culturing in an incubator at 37°C with 5% CO_2_, the cells were separated from the former medium. With a series of dosage concentrations of *P. polyphylla* var. *yunnanensis* extracted (11.25, 22.5, and 45 µM) from three different origins added into the wells, the plates were incubated for 24 h. Three parallel groups were set for each concentration, in addition to a group of the blank control. The obtained cells were fixed with 0.5 mL of the Hoechst reagent for 10 min, washed, and incubated in the darkness for 5 min. We discard the dye solution and place the sliver with the cellular side on a slide with an anti-fluorescent sealer. The prepared sample was observed under a fluorescence microscope at 350 nm (Olympus Corporation).

### 2.7 Flow cytometry analysis

The cell cycle was analyzed by flow cytometry using propyl iodide DNA staining. Propidium iodide (PI) is a cell-impermeant DNA binding dye that can be used to stain cells and nucleic acids. The cells were seeded in a number of 6-well plates at a density of 3×10^5^ cells/well and were then incubated in an incubator for 24 h. After the removal of the medium, the cells were respectively treated with dosage concentrations of 11.25, 22.5, and 45 µM of *P. polyphylla* var. *yunnanensis* extracted from three different origins for 24 h. After being washed with PBS, the cells were fixed with ice-cold 70% ethanol and then resuspended with 0.5 mL of propidium iodide. Finally, the cells were incubated for 30 min in the darkness at 37°C. The percentage of apoptotic cells was determined through flow cytometry, and the data were analyzed using FlowJo software.

### 2.8 *In vivo* animal experiment

Female BALB/c mice were purchased from Hunan Slyke Jingda Laboratory Animal Co., Ltd. The experiments were carried out with permission from the Institutional Animal Care and Use Committee (License number: SCXK (Xiang) 2019–0004). To construct a breast cancer model, 4T1 cells were injected subcutaneously into the armpit of mice at the density of 2 × 10^6^ cells/mL. Then, the mice were randomly divided into eight groups, namely, C1 low-dose group, C1 high-dose group, C2 low-dose group, C2 high-dose group, C3 low-dose group, C3 high-dose group, model group, and the positive drug Taxol group. The treatment was performed as follows. First, the mice in the three low-dose groups received i. p. injection of the extracts of *P. polyphylla* var. *yunnanensis* at a dosage of 75 mg/kg/day. Second, the mice in the three high-dose groups received i. p. injection of the extracts of *P. polyphylla* var. *yunnanensis* at a dosage of 300 mg/kg/day. Third, the mice in the positive group received i. p. injection of Taxol at a dosage of 10 mg/kg/day. Lastly, the mice in the model group received i. p. injection of the solvent on a daily basis. Before being euthanized by cervical dislocation, the mice were starved for 12 h.

### 2.9 Record tumor volume

The state of tumor growth was closely monitored with an electronic caliper at a 3-day interval after the tumor became observable using the formula V = a × b^2^/2, where a and b indicate the maximum and minimum superficial diameters, respectively.

### 2.10 TUNEL staining

TUNEL assay was employed to detect apoptosis in breast cancer tissues. The tissue samples were sliced to a size of 4 mm and subjected to pre-treatment. The ApopTag Peroxidase *In Situ* Apoptosis Detection Kit (Millipore, CA) was applied to perform the detection (Liu et al., 2017).

### 2.11 Immunohistochemical analysis

The samples of breast cancer tissues were sliced to a size of 4 mm and pre-treated. Then, the samples were fixed with formalin and immersed in paraffin. After 20 min of heat-induced antigen retrieval at 95°C, the serial sections were incubated overnight at 4°C with a combination of rabbit monoclonal anti-human p53 antibody (1:50 dilution; Abcam), rabbit monoclonal anti-human Ki67 antibody (1:500 dilution; Abcam), and rabbit monoclonal anti-human HER2 antibodies (1:500 dilution; Abcam). The Ki67, p53, and HER2 assays were performed as instructed by the manufacturer. Images were collected using NanoZoomer Digital Pathology 2 (Hamamatsu), and ImageJ software was used to quantitatively analyze the staining signals captured from six different images.

## 3 Results

### 3.1 The heavy metal content in *P. polyphylla* var. *yunnanensis*

**FIGURE 1:**
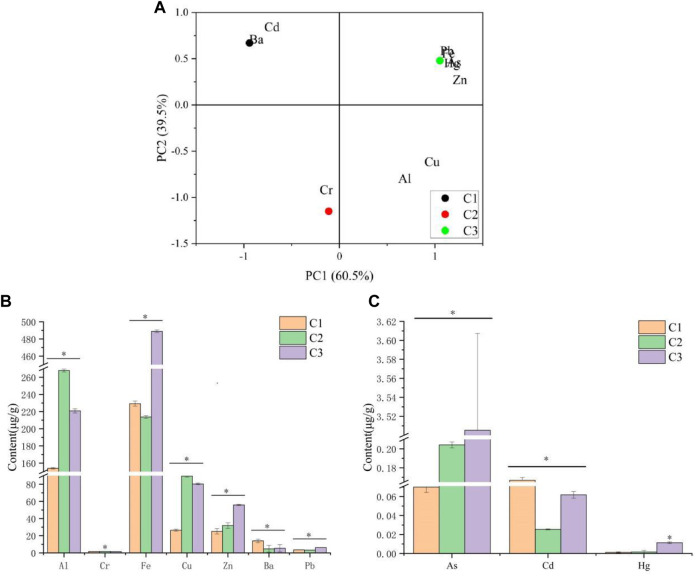
Heavy metal in *P. polyphylla* var. *yunnanensis*. **(A)** Correlation analysis between 10 heavy metals in *P. polyphylla* var. *yunnanensis* from three different origins. **(B)** Content of Al, Cr, Fe, Cu, Zn, Ba, and Pb in *P. polyphylla* var. *yunnanensis* from three different origins. **(C)** Content of As, Cd, and Hg in *P. polyphylla* var. *yunnanensis* from three different origins. The results are presented as mean ± SD, *n* = 3. *p <0.05.

The heavy metal present in *P. polyphylla* var. *yunnanensis* was detected by ICP-MS. According to the detection results of ICP-MS, Al, Cr, Fe, Cu, Zn, Ba, Pb, As, Cd, and Hg existed in *P. polyphylla* var. *yunnanensis*. Furthermore, the concentrations of Fe, Al, and Cu were high, while those of As, Cd, and Hg were low. There were significant differences in the contents of 10 kinds of heavy metals among three different origins. As can be seen from Figure 1A, there was a relatively large distance between the samples collected from the three different origins. Additionally, the content of Al, Fe, and Cu was higher, while that of Hg was the lowest, as shown in Figures 1B,C. The overall content of 10 metals in the three producing origins was in the following order: C3 >C2 >C1.

### 3.2 The steroidal saponin content in *P. polyphylla* var. *yunnanensis*

**FIGURE 2:**
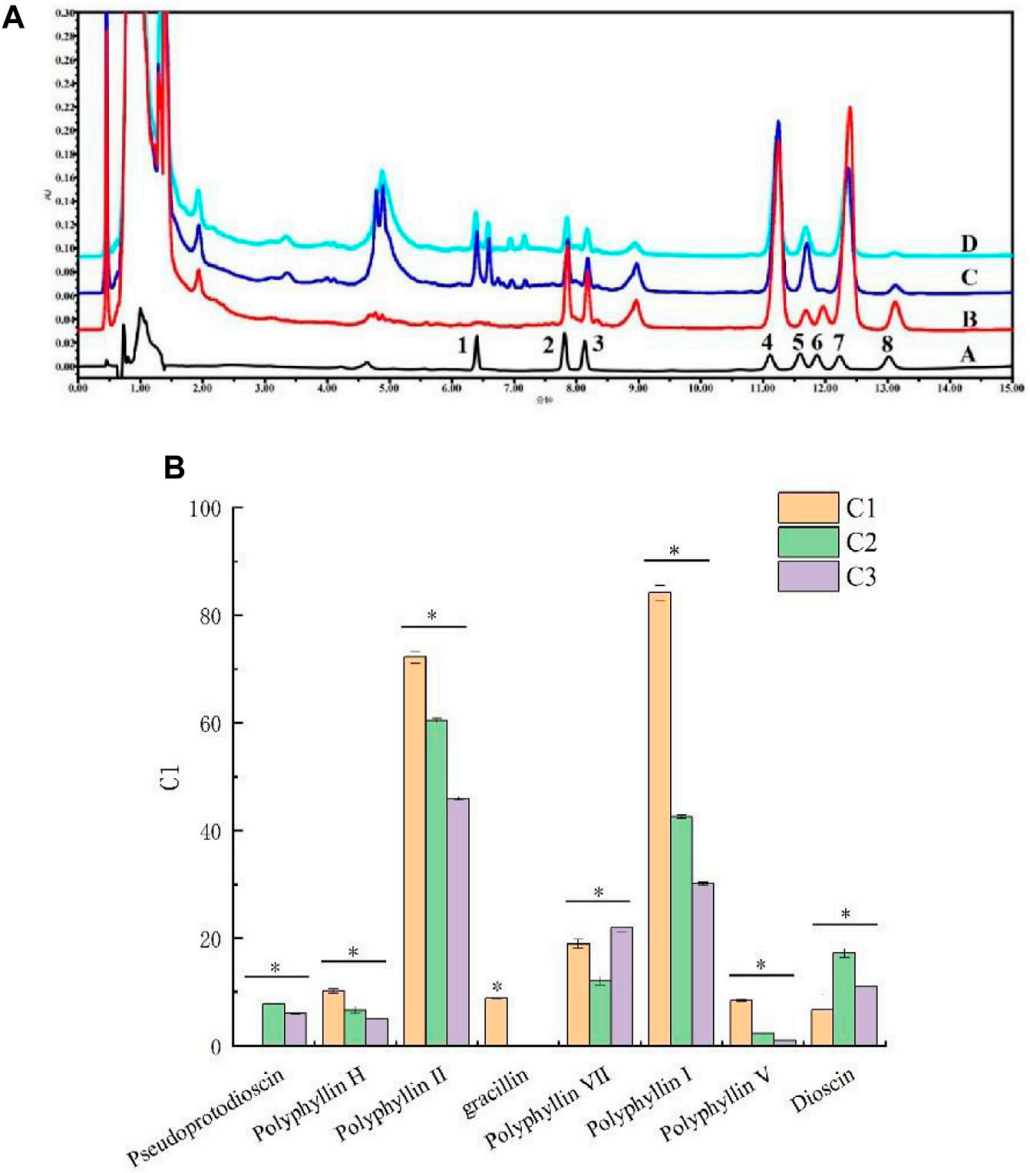
Steroidal saponin content in *P. polyphylla* var. *yunnanensis*. **(A)** HPLC chromatogram of eight varieties of steroid saponins in *P. polyphylla* var. *yunnanensis* (A: reference solution; B: C1 origin; C: C2 origin; D: C3 origin; 1. pseudoprotodioscin, 2. polyphyllin H, 3. polyphyllin II, 4. gracillin, 5. polyphyllin Ⅶ, 6. polyphyllin Ⅰ, 7. polyphyllin Ⅴ, and 8. dioscin); **(B)** content of the eight varieties of steroid saponins in *P. polyphylla* var. *yunnanensis*. The results are presented as mean ± SD, *n* = 3. *p <0.05.

The content of eight saponins in *P. polyphylla* var. *yunnanensis* samples was detected by HPLC, and the chromatogram is presented in Figure 2A. As shown in Figure 2B, there were significant differences observed in the content of eight saponins present in the extracts of *P. polyphylla* var. *yunnanensis* from three different origins (*p* <0.05). Among them, the pseudoprotodiosgenin from C2 origin and diosgenin from C1 and C3 origins were not detected. Among the eight varieties of saponins, the content of saponin Ⅰ and Ⅱ was found to be higher, followed by that of saponin Ⅶ and diosgenin. Furthermore, the saponin Ⅴ content was found to be lower than that of other saponins. The total content of eight saponins in the extracts of *P. polyphylla* var. *yunnanensis* from three different origins was in the following order: C1 >C2 >C3.

### 3.3 Correlation analysis of the heavy metal content and saponin content

**FIGURE 3:**
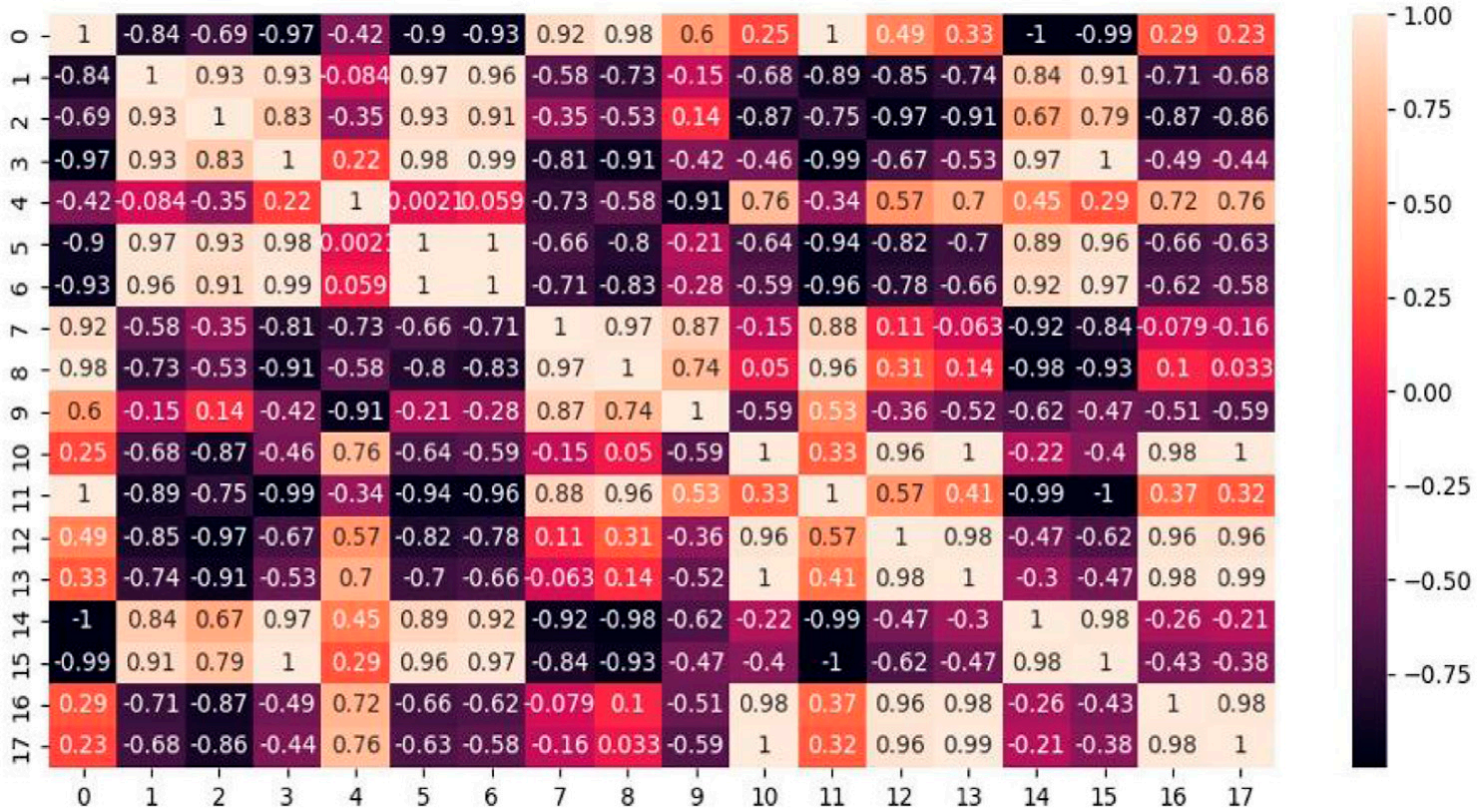
Pearson correlations between saponins and heavy metal content (0: pseudoprotodioscin; 1: polyphyllin H; 2: polyphyllin II; 3: gracillin; 4: polyphyllin Ⅶ; 5: polyphyllin Ⅰ; 6: polyphyllin Ⅴ; 7: dioscin; 8: Al; 9: Cr; 10: Fe; 11: Cu; 12: Zn; 13: As; 14: Cd; 15: Ba; 16: Hg; and 17: Pb).

Correlation analysis was carried out to assess the correlation between the contents of eight saponins and 10 heavy metals in *P. polyphylla* var. *yunnanensis* from C1 origins. The results are shown in Figure 3, it can be seen from the figure that elements Cd and Ba were positively correlated with the main steroid saponins in *P. polyphylla* var. *yunnanensis*, while elements Al, Cr, Fe, Cu, Zn, As, Hg, and Pb showed a negative correlation.

### 3.4 Effect of *P. polyphylla* var. *yunnanensis* from different origins on the proliferation of 4T1 cells

The CCK-8 assay was performed to examine the effects of *P. polyphylla* var. *yunnanensis* from different origins on the proliferation of 4T1 cells, the results of which are listed in Table 1. The extract of *P. polyphylla* var. *yunnanensis* from the three different regions, which inhibited the proliferation of 4T1 cells, showed a notable sign of concentration-dependent inhibitory effects within 48 h. Furthermore, the higher the concentration, the more significant the inhibitory effect on cell proliferation. When the concentration reached 45, 90, and 180 μM, there were significant variations observed in the inhibition rate of cells between these three different origins (*p* <0.05), with the inhibition rate in the following order: C1 >C3 >C2. The IC_50_ of *P. polyphylla* var. *yunnanensis* extracts from the origins C1, C2, and C3 was 74.63, 89.72, and 83.44 μM at 24 h and 14.26, 15.41, and 12.62 μg mL^-1^ at 48 h, respectively. For the subsequent apoptosis assay and flow cytometry assay, 45, 22.5, and 11.25 μg mL^-1^ were selected with a 24-h cell survival rate of more than 60%.

**TABLE 1:** Results of the proliferation rate of 4T1 cells of *P. polyphylla* var. *yunnanensis* from different origins.

Concentration (μg·mL^−1^)	Proliferation rate (%)					
	24 h			48 h		
	C1	C2	C3	C1	C2	C3
11.25	95.719 ± 1.499^a^	95.252 ± 1.691^a^	92.446 ± 0.324^a^	51.437 ± 0.062^a^	50.296 ± 0.327^a^	48.980 ± 0.234^a^
22.5	82.206 ± 0.360^a^	88.213 ± 1.835^a^	85.312 ± 2.68^a^	42.945 ± 0.538^a^	45.359 ± 1.69b^a^	41.540 ± 1.167^a^
45	61.511 ± 2.328^a^,^b^	74.676 ± 2.84^a^	78.106 ± 3.589^a^	35.177 ± 1.364^a^	39.258 ± 0.265^a^,^c^	31.731 ± 1.89^a^△
90	54.904 ± 2.328^a^,^b^	62.278 ± 0.359^a^,^b^	50.456 ± 1.491^a^,^b^	20.255 ± 2.596^a^	36.691 ± 1.21^a^,^b^	19.399 ± 0.271^a^
180	14.245 ± 2.492^a^	7.134 ± 2.753^b^	14.436 ± 0.577^a^	9.173 ± 0.031b^a^	7.004 ± 1.537^a^	6.715 ± 2.396^a^

^a^ Indicates a significant difference between 24 h and 28 h in different origins when *p* <0.05.

^b^ Indicates a significant difference with the other two different origins when *p* <0.05, given the same administration time.

^c^ Indicates that there is a significant difference between the two different origins when *p* <0.05, given the same administration time.

### 3.5 Effects of *P. polyphylla* var. *yunnanensis* from different origins on 4T1 cell apoptosis

**FIGURE 4:**
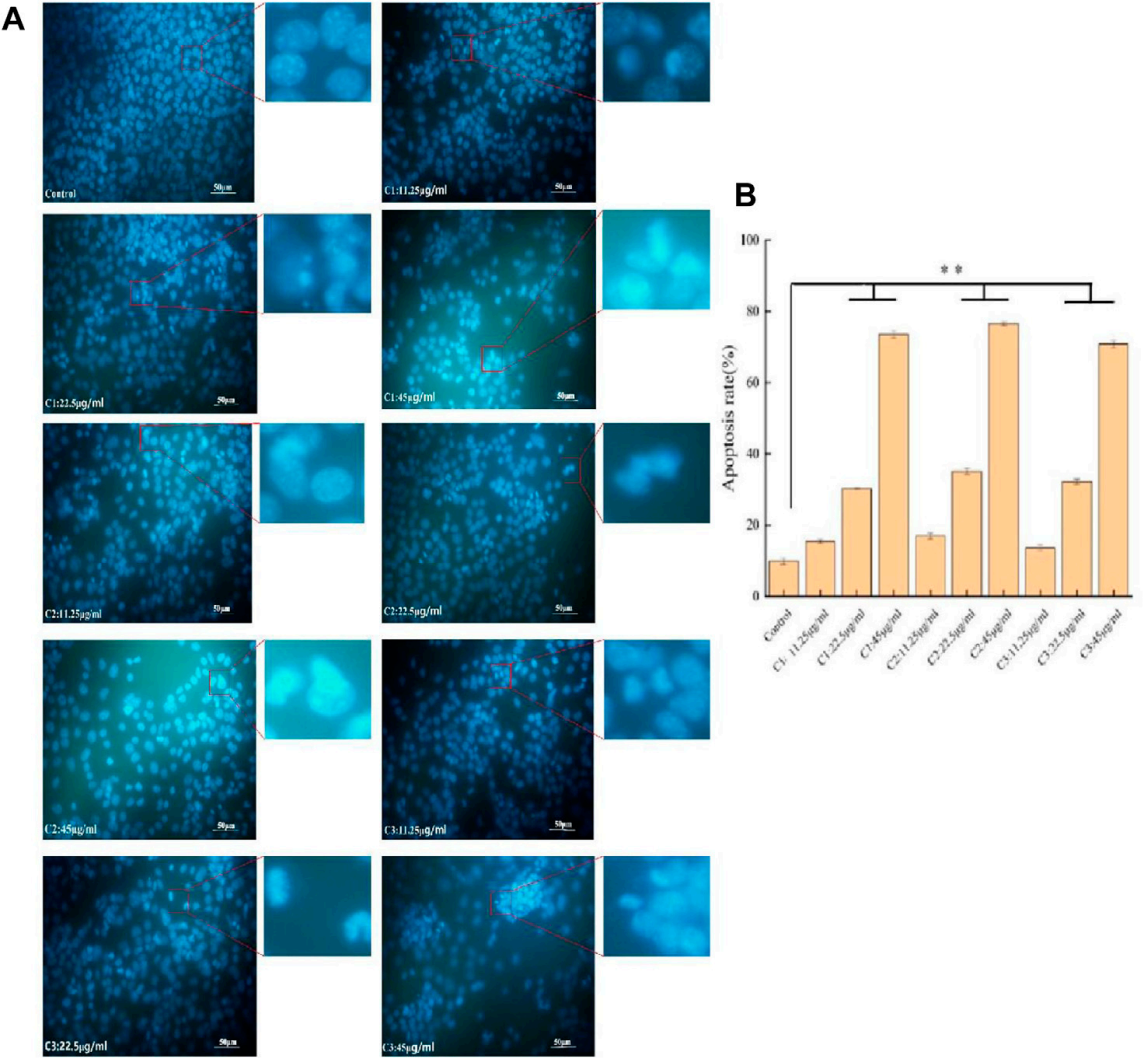
Effects of *P. polyphylla var. yunnanensis* from different origins on the apoptosis of 4T1 cells (×400; *n* = 6). **(A)** Images of Hoechst 33258 staining; **(B)** images of the apoptosis rate. The results are presented as mean ± SD, *n* = 6. **p <0.01.

Hoechst 33258 staining was performed to observe the apoptosis of 4T1 cells as induced by the extracts from three different origins, the results of which are shown in Figures 4A,B. In the control group, the level of cell fluorescence intensity was low and the distribution of cell fluorescence was uniform. Differently, the cell fluorescence intensity and chromatin condensation increased gradually in the drug treatment group with the increase of the drug concentration, showing signs of dense staining. Meanwhile, there were some differences found in the number of apoptotic cells, given the same concentration gradient among different origins.

### 3.6 Effect of *P. polyphylla* var. *yunnanensis* from different origins on the cell cycle of 4T1

**FIGURE 5:**
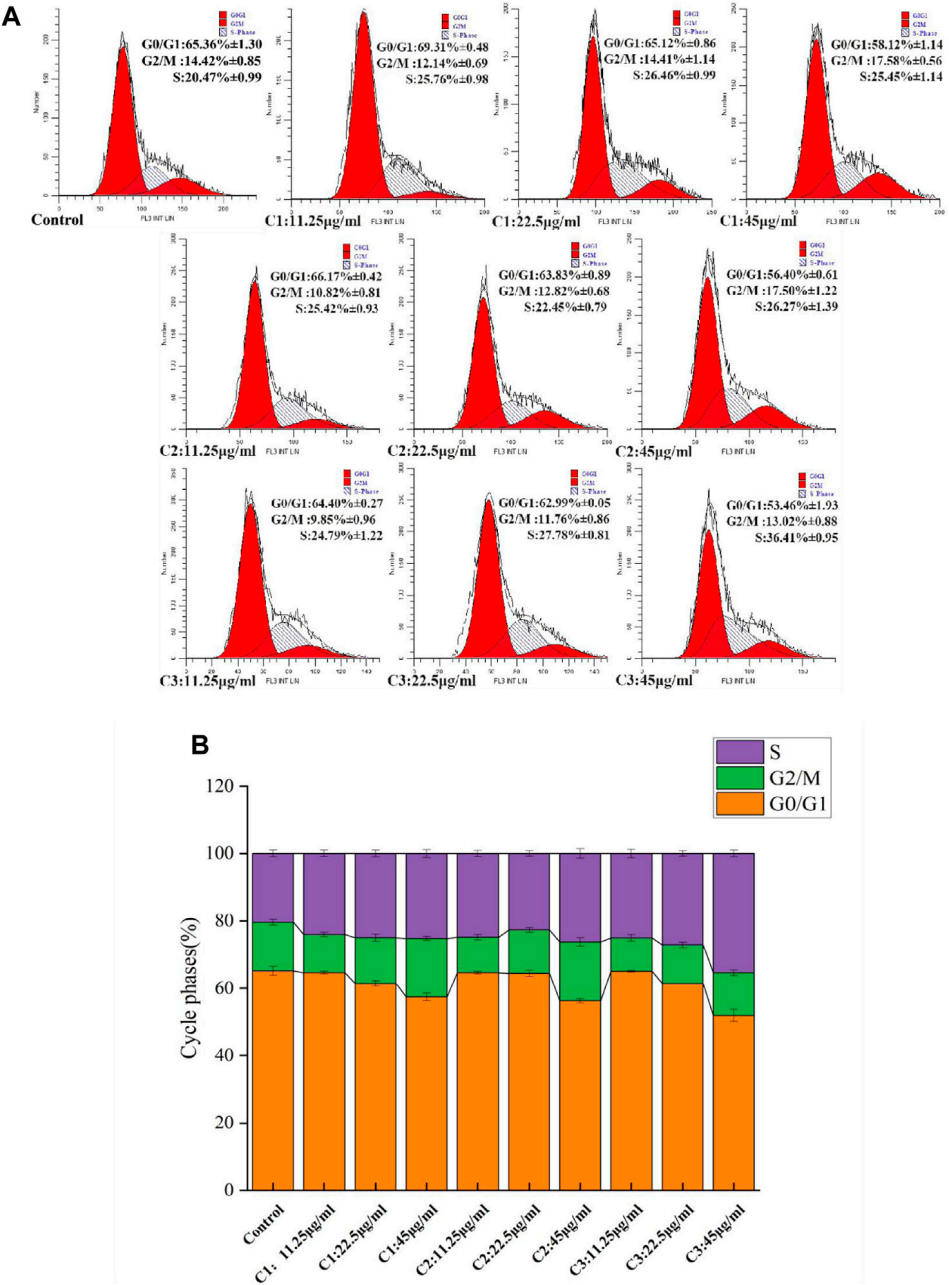
Effects of *P. polyphylla var. yunnanensis* from different origins on the 4T1 cell cycle. **(A)** Images of flow cytometry; **(B)** images of the cycle phases. The results are presented as mean ± SD, *n* = 6.

Figures 5A,B show the effects of *P. polyphylla* var. *yunnanensis* from different origins on the 4T1 cell cycle. When compared with the control group, the ratio of 4T1 cells in the G0/G1 phase declined gradually, while the ratio in the S phase increased progressively. By contrast, the ratio in the G2/M phase showed no significant change with the increase of the administration concentration. At the same time, there was no significant difference observed in the ratio of three different origins in the three periods (*p* >0.05). It is suggested that cell apotheosis was achieved by blocking the S phase in the three different origins.

### 3.7 Inhibited tumor growth in the 4T1-tumor mice

**FIGURE 6:**
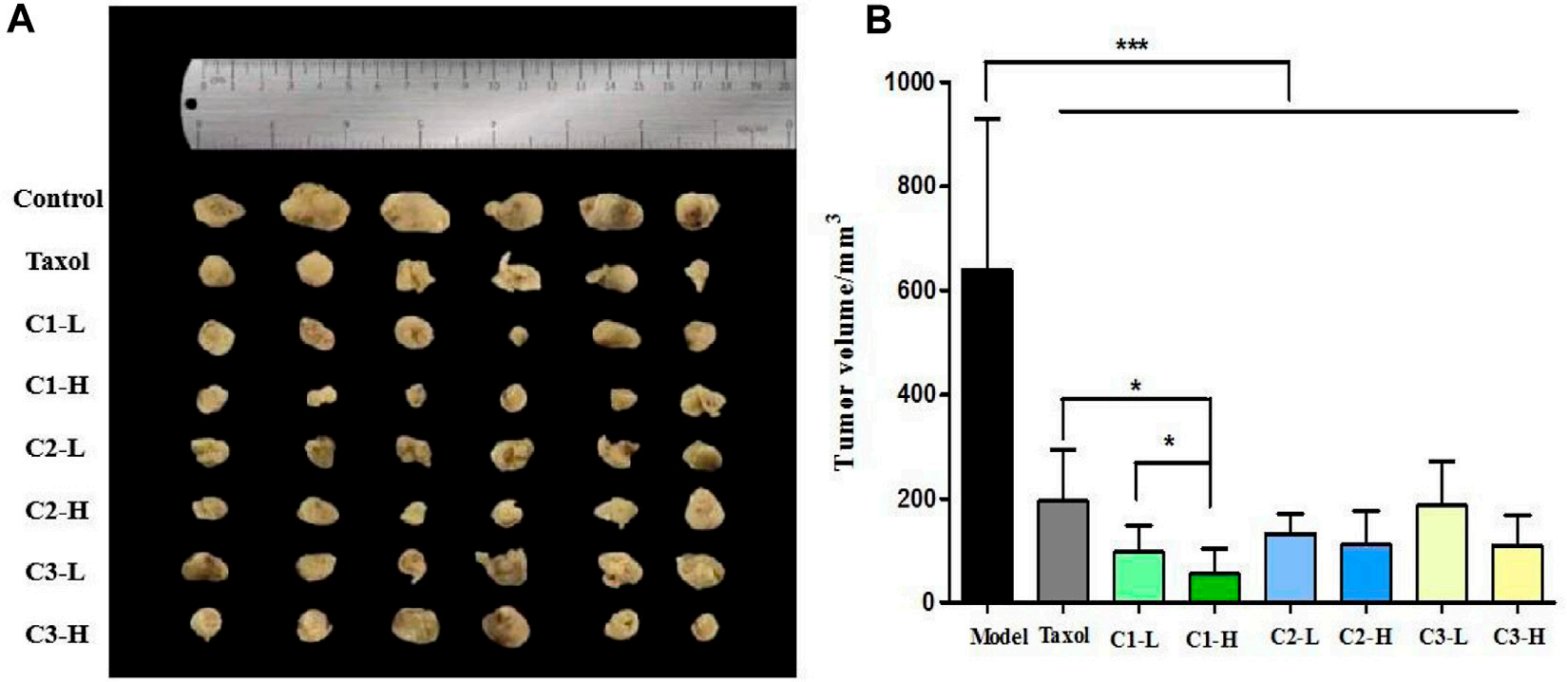
Effect of *P. polyphylla var. yunnanensis* from different origins on the tumor volume of 4T1-tumor mice. **(A)** Images of the tumors in 4T1-tumor mice; **(B)** tumor volume of 4T1-tumor mice. The results are presented as mean ± SD, *n* = 6. *p <0.05 and ***p <0.001.

Figures 6A,B show the effects of *P. polyphylla* var. *yunnanensis* from different origins on the tumor volume during treatment for the mice with breast cancer. According to the results, both Taxol and *P. polyphylla* var. *yunnanensis* extracts from different origins suppressed tumor growth when compared with the model group (*p* <0.001). These results demonstrate that *P. polyphylla* var. *yunnanensis* of different origins performed well in inhibiting tumor growth for the mice with breast cancer. The tumor inhibitory effect was more significant for high–low dose breast cancer mice in group C1 than for those in the other groups. Compared with the other groups, the inhibitory effect against the tumor was more evident in the Taxol group (*p* <0.05).

### 3.8 Effect on cancer cell proliferation and apoptosis induction in 4T1-tumor mice

**FIGURE 7:**
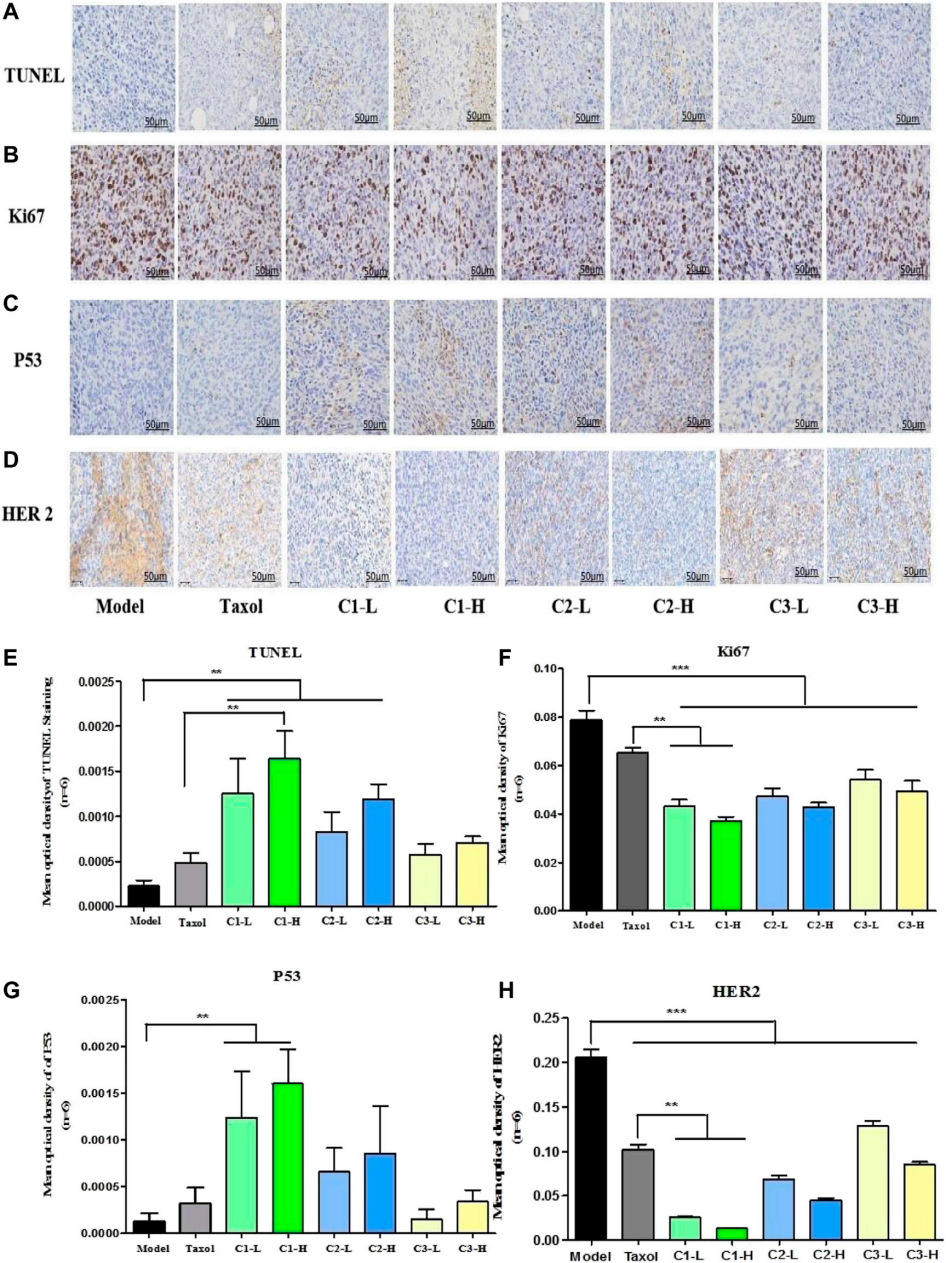
Effect of *P. polyphylla* var. *yunnanensis* from different origins on cancer cell proliferation and apoptosis induction in 4T1-tumor mice. **(A)** TUNEL staining (×400; *n* = 6). **(B)** Immunohistochemistry staining graph of Ki67 (×400; *n* = 6). **(C)** Immunohistochemistry staining graph of HER2 (×400; *n* = 6). **(D)** Immunohistochemistry staining graph of p53 (×400; *n* = 6). **(E)** Statistics of the TUNEL-positive cell. **(F)** Statistical analysis of Ki67 expression in 4T1-tumor mice. **(G)** Statistical analysis of p53 expression in 4T1-tumor mice. **(H)** Statistical analysis of p53 expression in 4T1-tumor mice. The results were expressed as mean ± SD, *n* = 6. **p <0.01 and ***p <0.001.

TUNEL assay was used to detect apotheosis in breast cancer tissues. The results of TUNEL staining showed that compared with the model group, the positive rate of apoptotic cells in transplanted tumor tissues of mice in the Taxol group and the *P. polyphylla* var. *yunnanensis* extract group from different origins was significantly increased (*p* <0.01), as shown in Figures 7A,E. The results show that the extract *P. polyphylla* var. *yunnanensis*-induced apoptosis in the transplanted tumor tissue of 4T1 mice. Among them, the high-dose group C1 showed the most significant apoptosis-inducing effect on the transplanted tumor tissue of 4T1 mice, when compared with the Taxol group (*p <*0.05).

Immunohistochemistry staining was used to observe the expression of Ki67, HER2, and p53 proteins in tumor tissues. Ki67 is an important indicator related to tumor proliferation. The higher the Ki67 positive proliferation, the faster the proliferation of tumor cells and the higher the aggressiveness of the tumor cells. HER2 is a membrane receptor, which is involved in regulating the proliferation of tumor cells. Overexpression of HER2 can lead to uncontrolled cell proliferation and tumor development, which is common in breast cancer and gastric cancer. The p53 tumor suppressor protein is a guard protein; when its expression is increased, it can promote the apoptosis of tumor cells. Compared with the model group, there was a significant decrease in the positive rates of Ki67 in the transplanted tumor tissues of mice from the paclitaxel group and the *P. polyphylla* var. *yunnanensis* extract from different origins (*p* <0.001) compared with the model group. Furthermore, compared with the Taxol group, the expression of Ki67 was significantly reduced in *P. polyphylla var. yunnanensis* of C1 origin (*p* <0.01), as shown in Figures 7B,F. In comparison with the model group, there was a sharp rise in the positive rates of p53 in the transplanted tumor tissues of mice from the paclitaxel group and the *P. polyphylla* var. *yunnanensis* extract from different origins (*p* <0.01), and there were no differences between *P. polyphylla var. yunnanensis* of other origins, as shown in Figures 7C,G. Compared with the Taxol group, the expression of HER2 was significantly reduced in *P. polyphylla* var. *yunnanensis* from C1 origin (*p* <0.01), as shown in Figures 7D,H. The results show that the *P. polyphylla* var. *yunnanensis* extract is applicable for reducing the expression of Ki67 and HER2, while improving the expression of the p53 protein in tumor tissues. The effect of the C1 producing origin is more significant relative to that of other producing origins.

## 4 Discussion

Toxic contents of heavy metals cause an enormous generation of reactive oxygen species (ROS) and damage to various biomolecules, leading to perturbation in their growth, physiology, and metabolism. However, the secondary metabolites are the chief defense strategies to overcome stress caused by heavy metals (Nasim and Dhir, 2010; Lajayer et al., 2017; Anjitha et al., 2021). In a study carried out for assessing the individual effect of Cu and Cd, they were found to enhance the content of phenolics, flavonoids, and saponins, hence showing the medicinal property of *Gynura procumbens* and had a higher antibacterial activity under Cd and Cu stress. In contrast, a combination of Cd and Cu caused a reduction in these metabolites, leading to reduced antibacterial activity (Ibrahim et al., 2017). Furthermore, some studies also reported the negative effect of heavy metals on secondary metabolites in medicinal plants. It was found that Cd stress reduced the accumulation of these saponins in *Panax notoginseng* as a result of the downregulated expression of phosphomevalonate kinase (PMK), mevalonate kinase (MVK), and geranylgeranyl diphosphate synthase (GGPS) of the terpenoid backbone biosynthesis pathway (Liao et al., 2019). The regulation of heavy metals on secondary metabolites of medicinal plants is bidirectional and indeterminate. In summary, heavy metal toxicity can alter its chemical makeup and have a significant impact on the quality, safety, and effectiveness of natural products made from this plant species.

With the fast-growing demand for *P. polyphylla* var. *yunnanensis*, as a type of raw material, the naturally available resources could not keep up with the market demand, which makes artificial planting a viable solution to the shortage of these resources. The present study focuses mainly on artificial planting, genetic diversity, structure, properties, and applications of *P. polyphylla* var. *yunnanensis*, despite some research studies revealing the effects of fungal elicitors on the secondary metabolite steroidal saponin in *P. polyphylla* var. *yunnanensis* (Zhou et al., 2004; Zhou et al. 2017; Zhou et al. 2017). However, there are still few studies exploring the effect of heavy metals of *P. polyphylla* var. *yunnanensis*, which has a significant impact on the content of active ingredients and pharmacological activity.

Focusing on the effects of heavy metals on the accumulation and pharmacological effects of active components in plants, the present study shows that steroid saponins are the main active components of *P. polyphylla* var. *yunnanensis*, revealing a close correlation between the heavy metal and the content of steroidal saponins. According to the Pearson correlation study, the content of Cd and Ba was positively correlated with the main steroidal saponins in *P. polyphylla* var. *yunnanensis*, while Al, Cr, Cu, Fe, Zn, As, Hg, and Pb showed a negative correlation. It is demonstrated that most of the heavy metal elements would adversely affect the accumulation of steroidal saponins in *P. polyphylla* var. *yunnanensis*, except that a few of them could promote the accumulation of active ingredients. However, the specific regulatory mechanisms of the effects of heavy metals on main steroidal saponins in *P. polyphylla* var. *yunnanensis* still need to be further studied.

It was also found that in the three origins, the content of heavy metals and steroidal saponins varied significantly between different origins. Origin C1 showed the lowest total heavy metal level but the highest total steroidal saponin level. Origin C2 showed a lower total heavy metal level and a lower total steroidal saponin level. Origin C3 showed the highest total heavy metal level but the lowest total steroidal saponin level. The effect on the pharmacological activities of anti-breast cancer of *P. polyphylla* var. *yunnanensis* from three different producing origins was further explored.

4T1 breast cancer cells were selected and cultivated for their antitumor studies *in vitro*. CCK-8 assay, Hoechst staining, and flow cytometry analysis were used for the examination of the proliferation and apoptosis of tumor cells. According to the results of the inhibitory effect on breast cancer antitumor experiment *in vitro*, the extracts of *P. polyphylla* var. *yunnanensis* from three origins inhibited the proliferation of 4T1 cells and induced cell apoptosis in a concentration- and time-dependent manner, especially in the C1 producing origin. The breast cancer 4T1 cells were subcutaneously injected into BALB/c mice to construct a tumor model to explore the *in vivo* inhibitory effect of *P. polyphylla var. yunnanensis* from three origins on breast cancer.

Mouse breast cancer 4T1 cells were subcutaneously injected into BALB/c mice to construct a tumor model *in vivo*. The results show that *P. polyphylla* var*. yunnanensis* extracts from different origins suppressed tumor growth when compared with the model group. The inhibitory effect of a high and low dose in the C1 group was more significant than that in the other groups. TUNEL assay was used to detect apotheosis, and immunohistochemistry staining was used to observe the expression of Ki67, HER2, and p53 proteins in breast cancer tissues. The extract of *P. polyphylla* var. *yunnanensis* could suppress the growth of tumors and induced the apoptosis of tumor cells. Meanwhile, they could reduce the expression of HER2 and Ki67 and enhance the expression of p53 in tumor tissues, especially in the C1 producing origin. In the three origins, origin C1 exhibits better antitumor pharmacological effects *in vivo* and *in vitro* than other origins.

## 5 Conclusion

The present study aims to reveal the characteristics of heavy metal contents, saponins, their antitumor pharmacological activity, and their interrelationships in *P. polyphylla* var. *yunnanensis* from different production areas. It is found that the content of saponins and its antitumor pharmacological activity decrease with the increase of heavy metal contents. The results show that the high content of total heavy metals may hinder the accumulation of steroidal saponins in *P. polyphylla* var. *yunnanensis* and result in the low biological activity. The results of this study suggest a necessity to control the content of heavy metals in the artificial cultivation process of *P. polyphylla* var. *yunnanensis*. To ensure the quality of artificial cultivation, future research should be conducted to explore the effects of changes in heavy metal morphology on the pharmacological activity given different land use patterns, which is essential for reasonably improving soil quality and the safety of *P. polyphylla* var. *yunnanensis* consumption.

## Data Availability

The original contributions presented in the study are included in the article/Supplementary Material; further inquiries can be directed to the corresponding authors.
